# Melatonin Alleviates Saline–Alkali Stress in Pakchoi by Protecting Photosynthetic Electron Transport, Ion Homeostasis, and Antioxidant Defense

**DOI:** 10.3390/biology15171506

**Published:** 2026-09-03

**Authors:** Baolong Du, Yongfu Ju, Jinbo Li, Yuan Wang, Juexian Dong, Jinlong Li, Nan Xu, Haixiu Zhong

**Affiliations:** 1 Heilongjiang Province Key Laboratory of Cold Region Wetland Ecology and Environment Research, School of Geography and Tourism, Harbin University, Harbin 150086, China; 2 Institute of Natural Resources and Ecology, Heilongjiang Academy of Sciences, Harbin 150040, China

**Keywords:** *Brassica rapa* subsp. *chinensis*, melatonin, saline–alkali stress, photosynthetic electron transport, chlorophyll fluorescence, ion homeostasis, antioxidant defense

## Abstract

Saline–alkali stress severely restricts leafy vegetable growth by impairing photosynthesis, ion homeostasis, and oxidative balance. This study investigated whether exogenous melatonin could alleviate saline–alkali stress in pakchoi (*Brassica rapa* subsp. *chinensis*) through coordinated protection of photosynthetic electron transport, ionic balance, and antioxidant defense. Pakchoi plants were subjected to four treatments: normal nutrient solution (CK), 100 μM melatonin (MT), 100 mM mixed saline–alkali stress with NaCl:NaHCO_3_ at 2:1 (SAS), and saline–alkali stress plus melatonin (SAS+MT). After 7 d, growth traits, gas exchange, chlorophyll fluorescence, OJIP transients, ion contents, osmotic adjustment, oxidative damage, and antioxidant enzyme activities were analyzed. Saline–alkali stress markedly inhibited growth, reduced photosynthetic pigment contents and gas exchange, impaired PSII photochemical performance, disrupted ion balance, and increased ROS accumulation and membrane damage. Compared with SAS, SAS+MT increased net photosynthetic rate (Pn) by 66.2%, effective quantum yield of PSII [Y_(II)_] by 56.0%, electron transport rate (ETR) by 57.9%, and the performance index on absorption basis (PI_ABS_) from 0.80 to 1.80. Melatonin also reduced Na^+^ content by 38.7%, increased the K^+^/Na^+^ ratio from 1.0 to 2.2, and enhanced superoxide dismutase (SOD),peroxidase (POD), catalase (CAT), and ascorbate peroxidase (APX) activities by 20.5%, 24.4%, 29.2%, and 33.3%, respectively. These results indicate that melatonin alleviates saline–alkali injury in pakchoi by maintaining PSII electron transport, improving ion homeostasis, and strengthening antioxidant defense.

## Simple Summary

Saline–alkali stress can restrict vegetable growth by disrupting water status, photosynthesis, ion homeostasis, and oxidative balance. In this study, we examined whether exogenous melatonin could alleviate the effects of mixed saline–alkali stress in pakchoi plants. Saline–alkali stress markedly inhibited growth, reduced leaf relative water content and photosynthetic performance, disrupted ion homeostasis, and increased oxidative damage. Foliar application of melatonin partially alleviated these adverse effects by maintaining photosynthetic electron transport, reducing Na^+^ accumulation, increasing the K^+^/Na^+^ ratio, and enhancing antioxidant enzyme activities. These results suggest that melatonin can partially improve the short-term physiological tolerance of pakchoi plants to saline–alkali stress.

## 1. Introduction

The salinity and alkalinity of soil pose significant limitations to vegetable cultivation in both protected and open-field growing conditions. Salt-damaged soils affect plant water uptake, nutrient uptake, cellular ion balance and photosynthetic metabolism, which finally results in decreased growth and yield of crops [1,2]. Saline–alkali stress may be more harmful than neutral salt stress because plants experience osmotic stress, Na^+^ toxicity as well as high-pH injury concurrently. Such stresses normally combine to suppress photosynthesis, destabilize membranes and induce excessive accumulation of reactive oxygen species (ROS) [2,3,4]. Thus, enhancing saline–alkali resistance in leafy vegetables is essential to ensure the productivity of crops in saline areas of cultivation.

Pakchoi (*Brassica rapa* subsp. *chinensis*) is one of the most valuable leafy vegetables, both economically and nutritionally. Nevertheless, pakchoi and other *Brassica rapa* crops are vulnerable to salt-induced stress, particularly at the seedling stage. It has been previously reported that salt stress inhibits pakchoi growth, inhibits leaf photosynthesis, changes Na^+^ and K^+^ distribution and induces antioxidant responses [5,6,7,8]. Such results suggest that salt tolerance in pakchoi is not governed by a single trait, but by a combination of photosynthetic stability, ion homeostasis, osmoregulation and ROS scavenging. Nevertheless, the mechanism of the physiological reactions of pakchoi to mixed saline–alkali stress, particularly the aspect of the electron transport in photosynthesis and the balance of ions, is not well understood.

Photosynthesis is highly sensitive to salt and saline–alkali stress. Stress-induced stomatal closure can restrict CO_2_ availability, whereas non-stomatal damage can impair chloroplast function and photosynthetic electron transport [2,3]. The study of chlorophyll fluorescence is a valuable method for assessing alterations in photosystem II (PSII) during stress conditions. The indicators of F_v_/F_m_, Y _(II)_, ETR, qP and NPQ indicate the photochemical efficiency of PSII, electron transport, and energy dissipation [9,10]. Additionally, OJIP transients and JIP-test parameters can indicate further changes in the functioning of the PSII donor side and acceptor side, such as energy absorption, electron transport, and thermal loss [11,12,13,14]. These instruments can help establish whether saline–alkali stress mostly leads to reversible photoprotection or to more serious damage to the photosynthetic machinery.

Another hallmark of salt-related injury is ion imbalance. Excessive Na^+^ accumulation interferes with K^+^ uptake, enzyme function, membrane function, and cellular metabolism [15,16,17]. It is generally thought that the ability to limit the accumulation of Na^+^ and maintain a high K^+^/Na^+^ ratio is an important physiological indicator of salt tolerance [18,19,20]. With this kind of ionic disturbance, osmotic stress and oxidative injury are frequently seen in saline–alkali environments. The response of plants includes the production of compatible solutes, like proline and soluble sugars, or the activation of antioxidant enzymes, like SOD, POD, CAT, and APX [2,4]. Nonetheless, if there is an abundance of ROS produced beyond the scavenging capacity, lipid peroxidation and membrane leakage will be enhanced, and there will be additional inhibition of photosynthesis and growth.

Melatonin is one of the endogenous indoleamines with roles as a regulator of plant growth as well as a molecule involved in stress response [21,22,23]. External melatonin has been shown to enhance salt tolerance in plants by increasing antioxidant levels, controlling osmotic regulation, safeguarding the photosynthetic apparatus, and maintaining homeostasis of ions [24,25,26,27,28,29,30]. More recent reports have also indicated that melatonin could enhance photosynthesis and photoprotection under abiotic stress conditions [31,32,33]. Nevertheless, although the protective effects of melatonin under conventional salt stress have been widely studied, its physiological role in pakchoi under mixed saline–alkali stress remains less well characterized, particularly with respect to the coordinated responses of PSII electron transport, ion homeostasis, and antioxidant defense.

Accordingly, the present study examined whether exogenous melatonin could alleviate mixed saline–alkali stress in pakchoi plants through coordinated physiological responses involving photosynthetic electron transport, ion homeostasis, and antioxidant defense. Specifically, we examined whether melatonin could (i) alleviate saline–alkali-induced growth inhibition and maintain leaf relative water content; (ii) preserve photosynthetic pigments, gas exchange, and PSII electron transport; (iii) improve Na^+^, K^+^, and Ca^2+^ balance and osmotic adjustment; and (iv) reduce oxidative injury by enhancing antioxidant enzyme activity. We hypothesized that melatonin could alleviate saline–alkali injury in pakchoi through the maintenance of photosynthetic electron transport, modulation of ion homeostasis, and enhancement of antioxidant defense.

## 2. Materials and Methods

### 2.1. Plant Material and Growth Conditions

Pakchoi plants (*Brassica rapa* subsp. chinensis cv. Shanghaiqing) were used as the experimental material. Plants were raised from seeds germinated in 50-cell seedling trays containing a peat/perlite substrate mixture (3:1, *v*/*v*). Following emergence, the plants were cultivated in a controlled-environment chamber with a 12 h/12 h light/dark photoperiod, day/night temperatures of 25/18 °C, a photosynthetic photon flux density (PPFD) of 200 μmol m^−2^ s^−1^, and a relative humidity of 70–80%. Plants were irrigated twice per week with half-strength Hoagland nutrient solution prepared according to Hoagland and Arnon [34]. When the plants reached the four-leaf stage, uniformly sized plants were selected and transferred to plastic pots filled with the same peat/perlite substrate. After 7 d of acclimation, when the plants were approximately 28–30 days old after sowing, the saline–alkali and melatonin treatments were initiated.

Pakchoi was selected because salt-related stress has been shown to affect growth, photosynthetic performance, ion accumulation, and antioxidant responses in pakchoi and related *Brassica rapa* crops [5,6,7,8].

### 2.2. Experimental Design and Treatments

Melatonin was first dissolved in a small volume of ethanol and then diluted with distilled water to a final concentration of 100 μM. The final melatonin solution contained 0.1% (*v*/*v*) ethanol and 0.05% (*v*/*v*) Tween-20 as a surfactant. A corresponding solvent-control solution containing the same concentrations of ethanol and Tween-20 but no melatonin was prepared in parallel.

The experiment consisted of four treatment combinations: CK, normal nutrient solution plus foliar solvent control; MT, normal nutrient solution plus foliar application of 100 μM melatonin; SAS, saline–alkali stress plus foliar solvent control; and SAS+MT, saline–alkali stress plus foliar application of 100 μM melatonin. Plants in the MT and SAS+MT treatments received the melatonin solution, whereas plants in the CK and SAS treatments received the corresponding solvent-control solution. No fixed spray volume per plant was predetermined. Instead, each solution was sprayed uniformly onto both the adaxial and abaxial leaf surfaces until the foliage was thoroughly wetted and visible runoff began, following a foliar application procedure used in previous melatonin studies [35]. The same spraying procedure and endpoint were used for all plants. Foliar treatments were applied 24 h before the start of saline–alkali treatment and again on the third day of stress treatment.

Saline–alkali stress was imposed using a mixed solution of NaCl and NaHCO_3_ at a molar ratio of 2:1, with a final total concentration of 100 mM. The pH of the mixed saline–alkali solution was 8.45 ± 0.10. The SAS and SAS+MT plants were irrigated with half-strength Hoagland nutrient solution containing the mixed salts, whereas CK and MT plants were irrigated with half-strength Hoagland nutrient solution without added salts. The treatment lasted for 7 d. Each treatment contained five biological replicates, and each biological replicate consisted of one independent plant. Plants were randomly arranged in the growth chamber and repositioned daily to minimize positional effects. At the end of the 7 d treatment period, the plants generally had approximately 9–12 true leaves per plant. Unless otherwise stated, the third to fourth fully expanded, non-senescent leaves from the shoot apex, representing leaves of comparable developmental status among plants, were used for physiological measurements and biochemical assays.

The mixed NaCl-NaHCO_3_ treatment has been applied to represent the combined effects of salinity and alkalinity that are frequently associated with osmotic stress, ion toxicity, injury due to high pH, oxidative stress, and inhibition of photosynthesis in plants [1,2,3,4].

### 2.3. Measurement of Growth Traits and Leaf Relative Water Content

The plant height, shoot fresh weight, shoot dry weight, leaf area, and leaf relative water content were measured at the conclusion of the 7 d treatment period. Plant height was measured by measuring the distance between the substrate surface and the highest point of growth on the plant with a ruler. Shoots were cut down and weighed instantly to determine their fresh weight. To measure the shoot dry weight, the samples were heated at 105 °C for 30 min, followed by drying at 75 °C until a constant weight was obtained.

The leaf area was determined using a portable leaf area meter LI-3000C (LI-COR Biosciences, Lincoln, NE, USA). Fresh weight (FW), turgid weight (TW) and dry weight (DW) were used to determine the leaf relative water content. The leaf discs were weighed immediately after excision to give FW, soaked in distilled water in darkness to give TW, and then oven-dried at 75 °C until a consistent weight was reached to give DW. Leaf relative water content was calculated as follows: RWC (%) = (FW − DW)/(TW − DW) × 100, with FW, TW and DW being fresh weight, turgid weight and dry weight respectively.

### 2.4. Photosynthetic Pigments and SPAD Values

SPAD values were measured using a SPAD-502 Plus chlorophyll meter (Konica Minolta, Osaka, Japan). Measurements were taken from the middle region of fully expanded leaves, avoiding the main vein. Five readings were taken from each biological replicate, and the average value was used for statistical analysis.

Photosynthetic pigments were determined using an acetone extraction method. Fresh leaf tissue (0.20 g) was cut into small pieces and extracted with 20 mL of 80% acetone in darkness until the tissue was completely decolorized. The extract was centrifuged at 10,000× *g* for 10 min, and the supernatant was used for absorbance measurement. Absorbance was recorded at 663, 646, and 470 nm using a UV-1800 UV–visible spectrophotometer (Shimadzu, Kyoto, Japan). Chlorophyll a, chlorophyll b, and carotenoid contents were calculated according to Lichtenthaler [36] and expressed as mg g^−1^ FW. An 80% acetone blank was used for absorbance correction.

### 2.5. Gas-Exchange Measurements

Leaf gas-exchange parameters were measured between 09:00 and 11:30 using an LI-6800 portable photosynthesis system (LI-COR Biosciences, Lincoln, NE, USA). Measurements were conducted on the third to fourth fully expanded leaves. The chamber conditions were set as follows: photosynthetic photon flux density, 1000 μmol m^−2^ s^−1^; CO_2_ concentration, 400 μmol mol^−1^; leaf temperature, 25 °C; and relative humidity, 60–70%.

Net photosynthetic rate (P*_n_*), stomatal conductance (G*_s_*), intercellular CO_2_ concentration (C*_i_*), and transpiration rate (T*_r_*) were recorded after the readings stabilized. Instantaneous water-use efficiency was calculated as:

WUE = P*_n_*/Tr

where P*_n_* is expressed as μmol CO_2_ m^−2^ s^−1^ and Tr is expressed as mmol H_2_O m^−2^ s^−1^. Gas-exchange measurements were interpreted following established principles for distinguishing stomatal and non-stomatal photosynthetic limitations [3,37].

### 2.6. Chlorophyll Fluorescence Measurements

Chlorophyll fluorescence parameters were measured using a PAM-2500 portable chlorophyll fluorometer (Heinz Walz, Effeltrich, Germany). Leaves were dark-adapted for 30 min before measurement. Minimum fluorescence (F_o_) was recorded under weak measuring light, and maximum fluorescence (F_m_) was obtained using a saturating light pulse of 8000 μmol m^−2^ s^−1^ for 0.8 s. The maximum quantum efficiency of PSII was calculated as:

F_v_/F_m_ = (F_m_ − F_o_)/F_m_

After dark-adapted measurements, leaves were exposed to actinic light at 350 μmol m^−2^ s^−1^. Steady-state fluorescence (F_s_) and maximum fluorescence under light (F_m_′) were recorded using saturating pulses. The effective quantum yield of PSII [Y_(II)_], electron transport rate (ETR), photochemical quenching coefficient (qP), and non-photochemical quenching (NPQ) were calculated according to standard chlorophyll fluorescence protocols [9,10,38]. ETR was calculated as:

ETR = Y_(II)_ × PPFD × 0.84 × 0.5

where PPFD was 350 μmol m^−2^ s^−1^ during fluorescence measurement, 0.84 represents the assumed leaf absorptance, and 0.5 represents the assumed equal distribution of absorbed light energy between PSI and PSII.

### 2.7. OJIP Transient and JIP-Test Analysis

Fast chlorophyll a fluorescence transients were recorded using a multifunctional plant efficiency analyzer M-PEA (Hansatech Instruments, Norfolk, UK). Leaves were dark-adapted for 30 min before measurement. Fluorescence induction curves were recorded under a saturating red light pulse of 3000 μmol m^−2^ s^−1^ for 1 s.

The OJIP transient was used to evaluate PSII photochemical performance. Relative variable fluorescence at the J step (V*_J_*) and the K-step-related parameter (W*_K_*) were calculated from the fluorescence transient. JIP-test parameters, including ABS/RC, TR_o_/RC, ET_o_/RC, DI_o_/RC, and PI*_ABS_*, were calculated according to the JIP-test framework [11,12,13,14]. ABS/RC represents the apparent antenna size per active PSII reaction center; TR_o_/RC represents trapped energy flux per reaction center; ET_o_/RC represents electron transport flux per reaction center; DI_o_/RC represents dissipated energy flux per reaction center; and PI*_ABS_* represents the performance index on an absorption basis.

### 2.8. Ion Content Determination

Leaf samples were oven-dried at 75 °C to constant weight and ground into fine powder. Approximately 0.10 g of dried sample was digested with 8 mL HNO_3_ and 2 mL H_2_O_2_ using a microwave digestion system (MARS 6, CEM Corporation, Matthews, NC, USA). After digestion, the solution was cooled, filtered, and diluted to 25 mL with deionized water.

Na^+^, K^+^, and Ca^2+^ concentrations were determined using an inductively coupled plasma optical emission spectrometer (ICP-OES, Agilent 5110, Agilent Technologies, Santa Clara, CA, USA). Ion contents were expressed as mg g^−1^ DW. The K^+^/Na^+^ ratio was calculated from the measured K^+^ and Na^+^ concentrations. Maintenance of a high K^+^/Na^+^ ratio is a key physiological feature of salt tolerance, whereas excessive Na^+^ accumulation disrupts cellular ion homeostasis and metabolic stability [15,16,17,18,19,20].

### 2.9. Osmotic Adjustment Substances

The acid ninhydrin technique was used to determine free proline content [39]. Fresh leaf sample (0.50 g) was homogenized with 3 percent sulfo salicylic acid and boiled in a boiling water bath for 10 min. The centrifuged supernatant was combined with acid ninhydrin reagent and glacial acetic acid and incubated at 100 °C. The reaction mix was extracted with toluene, and the absorbance of the solution was recorded at 520 nm. Proline content was determined by a standard curve and reported as μmol g^−1^ FW.

Soluble sugar content was determined using the anthrone–sulfuric acid method described by Yemm and Willis [40]. Fresh leaf tissue (0.50 g) was used for each determination. Extraction with distilled water and the subsequent anthrone–sulfuric acid color-development procedure were performed according to the published method. Absorbance was measured at 620 nm, and soluble sugar content was calculated from the standard curve and expressed as mg g^−1^ FW.

Leaf soluble protein content was determined using the Coomassie Brilliant Blue G-250 method [41]. Fresh leaf tissue was extracted with phosphate buffer, and the extract was reacted with Coomassie Brilliant Blue G-250 reagent. Absorbance was measured at 595 nm using bovine serum albumin as the standard. Soluble protein content was expressed as mg g^−1^ FW and analyzed as an osmotic adjustment-related physiological parameter.

### 2.10. Oxidative Damage and Antioxidant Enzyme Activities

The fresh leaves were harvested immediately after the measurement of physiological parameters and preserved under liquid nitrogen, and then kept at −80 °C until biochemical analysis. The protein concentration of the crude enzyme extract was determined using the Bradford method solely for normalization of antioxidant enzyme activities, which were expressed as U mg^−1^ protein [41].

Hydrogen peroxide (H_2_O_2_) content was determined using the titanium sulfate method described by Jana and Choudhuri [42]. Fresh leaf tissue was extracted, and the subsequent reaction procedure was performed according to the published method. After centrifugation at 12,000× *g* for 10 min at 4 °C, the resulting supernatant was reacted with potassium phosphate buffer and titanium sulfate solution. Absorbance was measured at 410 nm. An extinction coefficient of 0.28 mM^−1^ cm^−1^ was used, and H_2_O_2_ content was expressed as μmol g^−1^ FW.

The superoxide anion (O_2_^−^) production rate was determined using the hydroxylamine method described by Elstner and Heupel [43]. Fresh leaf tissue extraction and the subsequent hydroxylamine reaction and color-development procedures were performed according to the published method. The resulting extract was reacted sequentially with hydroxylamine hydrochloride, sulfanilic acid, and α-naphthylamine, and absorbance was measured at 530 nm. The O_2_^−^ production rate was expressed as μmol g^−1^ FW h^−1^.

Malondialdehyde (MDA) content was determined using the thiobarbituric acid method described by Heath and Packer [44]. Fresh leaf tissue extraction and the subsequent reaction procedure were performed according to the published method. The extract was reacted with 20% trichloroacetic acid containing 0.5% thiobarbituric acid, heated at 95 °C for 30 min, rapidly cooled, and centrifuged. Absorbance was measured at 532, 600, and 450 nm, and MDA content was expressed as nmol g^−1^ FW.

Relative electrolyte leakage (REL) was determined using the conductivity method described by Lutts et al. [45]. Leaf sampling and preparation were performed according to the published method. The leaf disks were washed with deionized water, immersed in 20 mL of deionized water, and incubated at room temperature for 12 h. The initial conductivity (C1) was then measured using a conductivity meter (DDS-307A, Leici, Shanghai, China). The samples were subsequently boiled for 20 min, cooled to room temperature, and the total conductivity (C2) was measured. REL was calculated as REL (%) = C1/C2 × 100.

For antioxidant enzyme extraction, 0.50 g of fresh leaf tissue was homogenized with 5 mL of ice-cold 50 mM potassium phosphate buffer (pH 7.0) containing 1 mM EDTA and 1% (*w*/*v*) polyvinylpolypyrrolidone. The homogenate was centrifuged at 12,000× *g* for 15 min at 4 °C, and the resulting supernatant was used as the crude enzyme extract. The protein concentration of the crude enzyme extract was determined using the Bradford method for normalization of enzyme activities [41].

Superoxide dismutase (SOD) activity was determined according to Beauchamp and Fridovich by measuring the inhibition of nitroblue tetrazolium photoreduction at 560 nm [46]. Peroxidase (POD) activity was determined according to Chance and Maehly using the guaiacol–H_2_O_2_ method by monitoring the increase in absorbance at 470 nm [47]. Catalase (CAT) activity was determined according to Aebi by monitoring the decrease in absorbance at 240 nm caused by H_2_O_2_ decomposition [48]. Ascorbate peroxidase (APX) activity was determined according to Nakano and Asada by monitoring the decrease in absorbance at 290 nm caused by ascorbate oxidation [49]. The reaction compositions and enzyme-activity calculations followed the respective published methods. Enzyme activities were expressed as U mg^−1^ protein.

### 2.11. Statistical Analysis

All data were expressed as mean ± SD based on five biological replicates. Statistical analyses were performed using SPSS 26.0. Data normality and homogeneity of variance were examined using the Shapiro–Wilk test and Levene’s test, respectively. Percentage variables, such as relative water content and relative electrolyte leakage, were transformed when necessary before analysis.

Because the experiment included two factors, saline–alkali stress and melatonin application, two-way ANOVA was first used to test the main effects of saline–alkali stress, melatonin, and their interaction. When significant treatment effects were detected, one-way ANOVA followed by Tukey’s honestly significant difference test was used to compare the four treatment means at *p* < 0.05.

## 3. Results

### 3.1. Growth Traits and Leaf Relative Water Content

**Figure 1:**
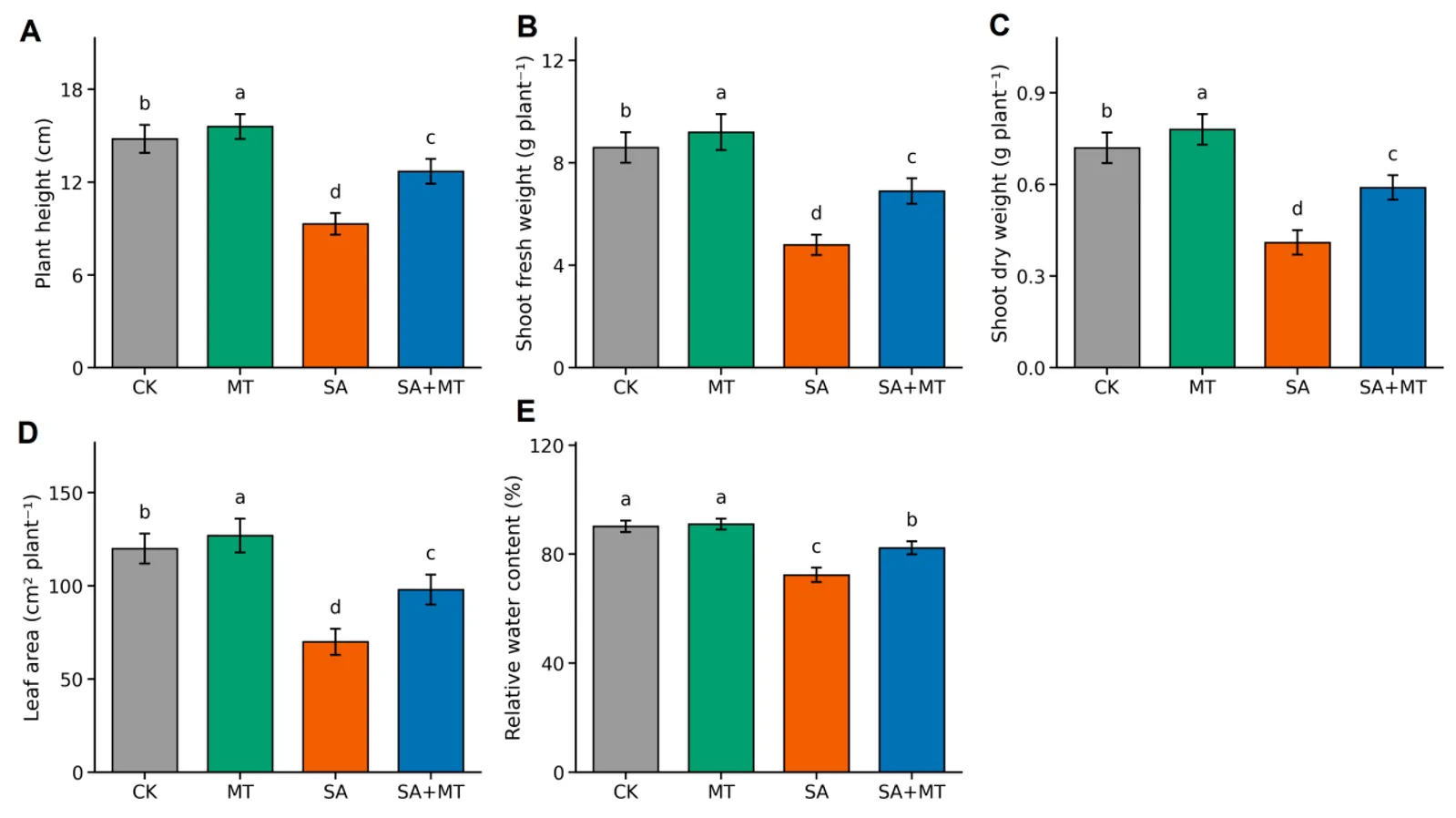
Growth traits and leaf relative water content of pakchoi under different saline–alkali stress and melatonin treatments. Values of (**A**) plant height, (**B**) shoot fresh weight, (**C**) shoot dry weight, (**D**) leaf area, and (**E**) leaf relative water content under the different treatments. CK, normal nutrient solution plus foliar solvent control; MT, normal nutrient solution plus foliar application of 100 μM melatonin; SAS, saline–alkali stress plus foliar solvent control; SAS+MT, saline–alkali stress plus foliar application of 100 μM melatonin. Values are means ± SD (*n* = 5). Different lowercase letters indicate significant differences among treatments according to Tukey’s HSD test at *p* < 0.05.

Compared with CK, SAS reduced plant height, shoot fresh weight, shoot dry weight, and leaf area by 37.2%, 44.2%, 43.1%, and 41.7%, respectively (Figure 1A–D). Leaf relative water content decreased from 90.2% in CK to 72.4% under SAS (Figure 1E).

Under non-stress conditions, MT did not significantly alter the measured growth traits compared with CK (*p* > 0.05). Under saline–alkali stress, melatonin significantly alleviated the reductions in growth traits. Compared with SAS, SAS+MT increased plant height, shoot fresh weight, shoot dry weight, and leaf area by 36.6%, 43.8%, 43.9%, and 40.0%, respectively. Leaf relative water content increased from 72.4% under SAS to 82.3% under SAS+MT.

### 3.2. Photosynthetic Pigment Contents and Gas-Exchange Parameters

**Figure 2:**
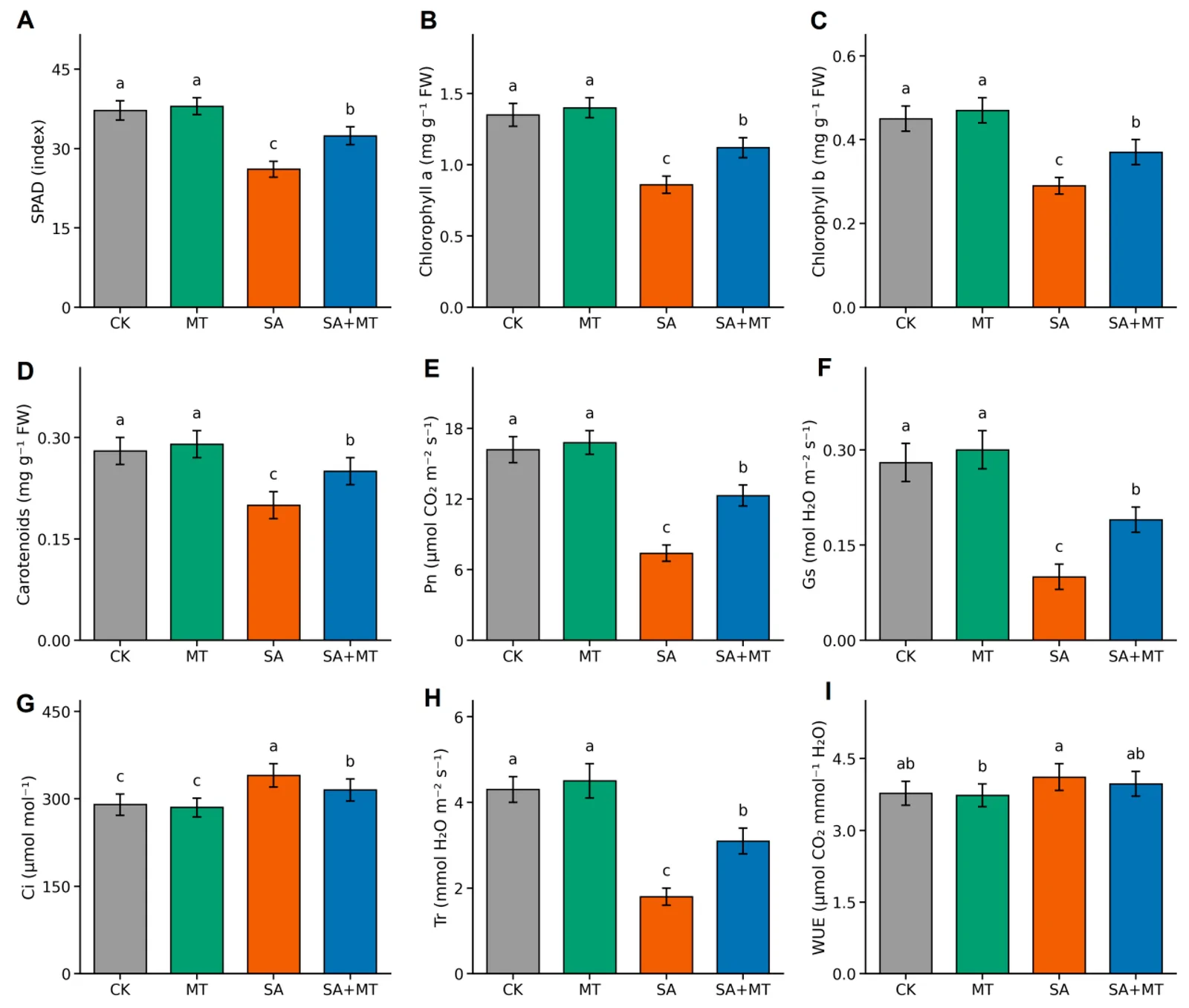
Photosynthetic pigment contents and gas-exchange parameters of pakchoi under different saline–alkali stress and melatonin treatments. Values of (**A**) SPAD, (**B**) chlorophyll a, (**C**) chlorophyll b, (**D**) carotenoids, (**E**) net photosynthetic rate (P_n_), (**F**) stomatal conductance (G_s_), (**G**) intercellular CO_2_ concentration (C_i_), (**H**) transpiration rate (T_r_), and (**I**) instantaneous water-use efficiency under the different treatments. CK, normal nutrient solution plus foliar solvent control; MT, normal nutrient solution plus foliar application of 100 μM melatonin; SAS, saline–alkali stress plus foliar solvent control; SAS+MT, saline–alkali stress plus foliar application of 100 μM melatonin. Values are means ± SD (*n* = 5). Different lowercase letters indicate significant differences among treatments according to Tukey’s HSD test at *p* < 0.05.

Saline–alkali stress significantly affected photosynthetic pigment-related traits. Compared with CK, SAS reduced the SPAD value by 29.8%, while chlorophyll a, chlorophyll b, and carotenoid contents decreased by 36.3%, 35.6%, and 28.6%, respectively (Figure 2A–D). Compared with SAS, SAS+MT increased the SPAD value by 24.1% and chlorophyll a, chlorophyll b, and carotenoid contents by 30.2%, 27.6%, and 25.0%, respectively.

Compared with CK, SAS decreased the net photosynthetic rate (P*_n_*), stomatal conductance (G_s_), and transpiration rate (T_r_) by 54.3%, 64.3%, and 58.1%, respectively (Figure 2E,F,H). In contrast, the intercellular CO_2_ concentration (C_i_) increased by 17.2% under SAS compared with CK (Figure 2G). Compared with SAS, SAS+MT increased P*_n_* by 66.2%, G_s_ by 90.0%, and T_r_ by 72.2%, while Ci decreased from 340 to 315 μmol mol^−1^. WUE showed moderate variation among treatments, with the highest value under SAS and intermediate values under SAS+MT (Figure 2I).

### 3.3. Chlorophyll Fluorescence Parameters

**Figure 3:**
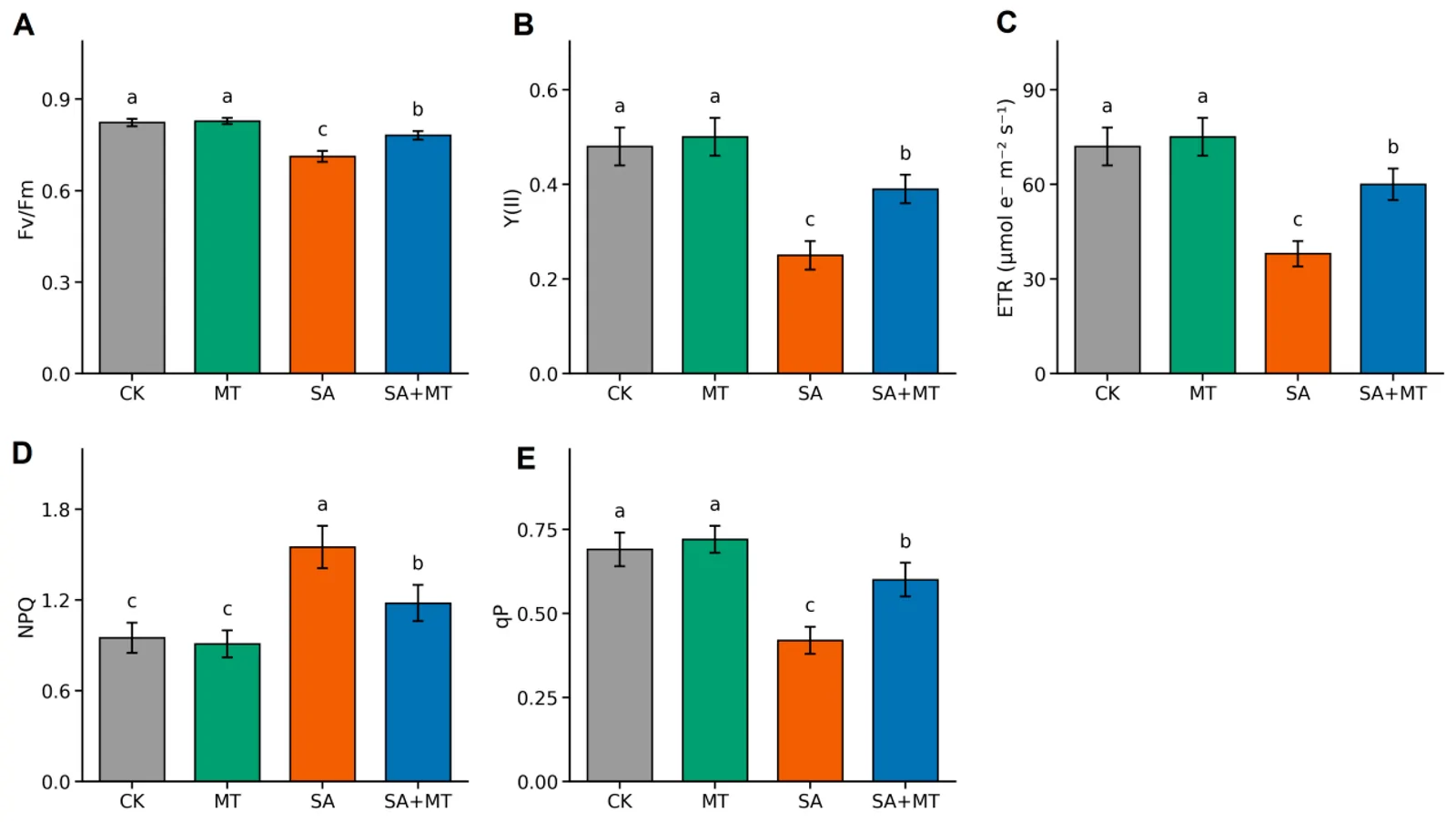
Chlorophyll fluorescence parameters of pakchoi under different saline–alkali stress and melatonin treatments. Values of (**A**) F_v_/F_m_, (**B**) Y_(II)_, (**C**) electron transport rate (ETR), (**D**) non-photochemical quenching (NPQ), and (**E**) photochemical quenching coefficient (qP) under the different treatments. CK, normal nutrient solution plus foliar solvent control; MT, normal nutrient solution plus foliar application of 100 μM melatonin; SAS, saline–alkali stress plus foliar solvent control; SAS+MT, saline–alkali stress plus foliar application of 100 μM melatonin. Values are means ± SD (*n* = 5). Different lowercase letters indicate significant differences among treatments according to Tukey’s HSD test at *p* < 0.05.

Saline–alkali stress significantly altered chlorophyll fluorescence parameters. Compared with CK, SAS decreased F_v_/F_m_ from 0.823 to 0.712, corresponding to a reduction of 13.5% (Figure 3A). Y_(II)_, ETR, and qP decreased by 47.9%, 47.2%, and 39.1%, respectively (Figure 3B,C,E), whereas NPQ increased by 63.2% (Figure 3D). Compared with SAS, SAS+MT increased F_v_/F_m_, Y_(II)_, ETR, and qP by 9.7%, 56.0%, 57.9%, and 42.9%, respectively, while NPQ decreased by 23.9%.

### 3.4. OJIP Transients and JIP-Test Parameters

**Figure 4:**
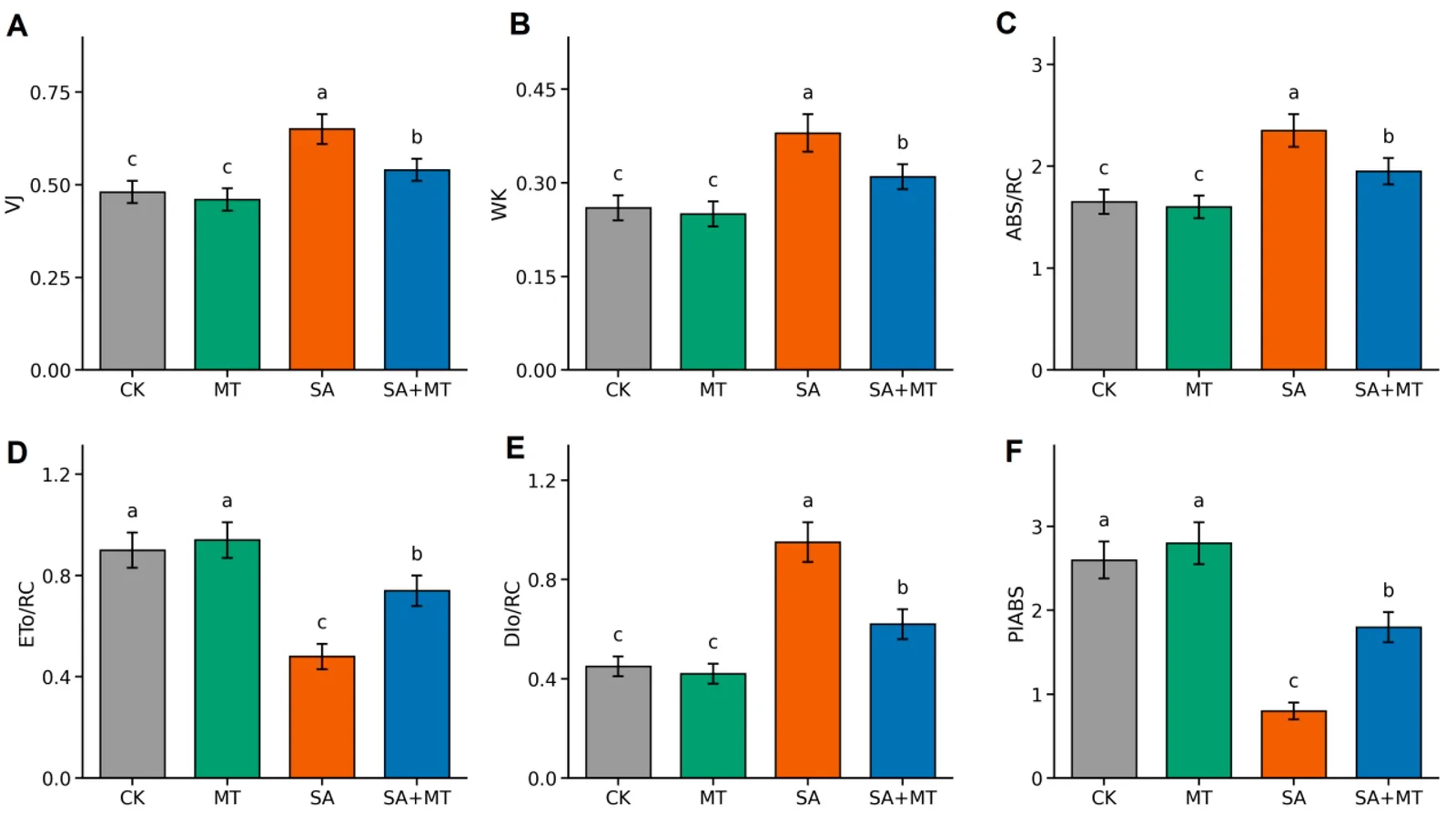
OJIP transients and JIP-test parameters of pakchoi under different saline–alkali stress and melatonin treatments. (**A**) Normalized OJIP chlorophyll a fluorescence transients and values of (**B**) VJ, (**C**) WK, (**D**) ABS/RC, (**E**) ET_o_/RC, (**F**) DIo/RC, under the different treatments. V_J_ represents the relative variable fluorescence at the J step; WK represents the relative fluorescence associated with the K step; ABS/RC, ET_o_/RC, and DIo/RC represent absorbed energy flux, electron transport flux, and dissipated energy flux per active PSII reaction center, respectively; PIABS represents the performance index on an absorption basis. CK, normal nutrient solution plus foliar solvent control; MT, normal nutrient solution plus foliar application of 100 μM melatonin; SAS, saline–alkali stress plus foliar solvent control; SAS+MT, saline–alkali stress plus foliar application of 100 μM melatonin. Values are means ± SD (*n* = 5). Different lowercase letters indicate significant differences among treatments according to Tukey’s HSD test at *p* < 0.05.

Saline–alkali stress significantly altered the OJIP transients and JIP-test parameters (Figure 4). Compared with CK, SAS increased VJ and WK by 35.4% and 46.2%, respectively (Figure 4A,B). SAS also increased ABS/RC and DIo/RC by 42.4% and 111.1%, respectively, while ET_o_/RC and PIABS decreased by 46.7% and 69.2%, respectively (Figure 4C–F). Compared with SAS, SAS+MT decreased VJ, WK, ABS/RC, and DIo/RC by 16.9%, 18.4%, 17.0%, and 34.7%, respectively. ET_o_/RC increased by 54.2%, while PIABS increased from 0.80 under SAS to 1.80 under SAS+MT.

### 3.5. Ion Contents and Osmotic Adjustment Parameters

**Figure 5:**
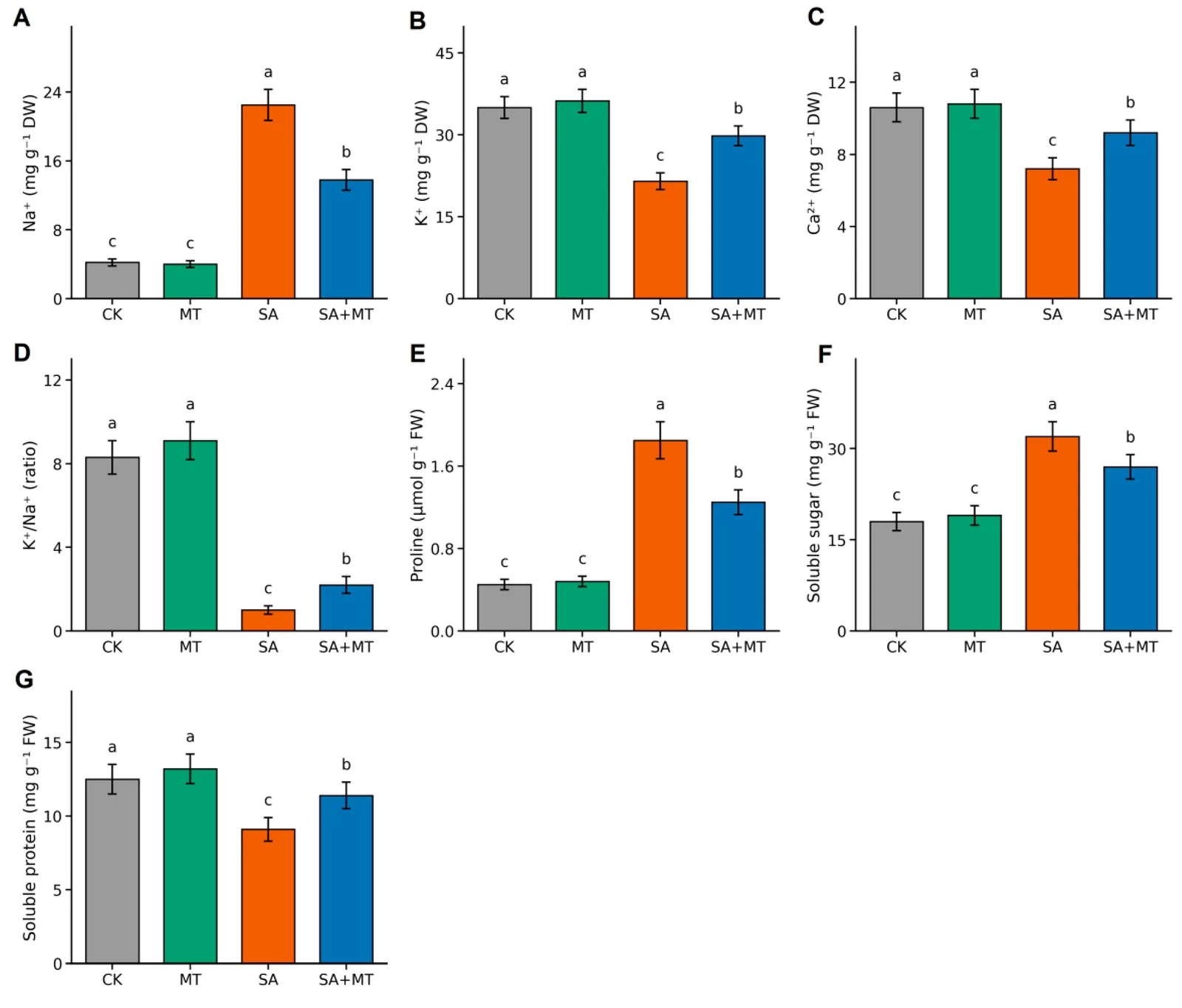
Ion contents and osmotic adjustment parameters of pakchoi under different saline–alkali stress and melatonin treatments. Values of (**A**) Na^+^ content, (**B**) K^+^ content, (**C**) Ca^2+^ content, (**D**) K^+^/Na^+^ ratio, (**E**) proline content, (**F**) soluble sugar content, and (**G**) soluble protein content under the different treatments. CK, normal nutrient solution plus foliar solvent control; MT, normal nutrient solution plus foliar application of 100 μM melatonin; SAS, saline–alkali stress plus foliar solvent control; SAS+MT, saline–alkali stress plus foliar application of 100 μM melatonin. Values are means ± SD (*n* = 5). Different lowercase letters indicate significant differences among treatments according to Tukey’s HSD test at *p* < 0.05.

Compared with CK, SAS increased Na^+^ content by 435.7%, whereas K^+^ and Ca^2+^ contents decreased by 38.6% and 32.1%, respectively (Figure 5A–C). The K^+^/Na^+^ ratio decreased from 8.3 in CK to 1.0 under SAS (Figure 5D). Compared with SAS, SAS+MT reduced Na^+^ content by 38.7% and increased K^+^ and Ca^2+^ contents by 38.6% and 27.8%, respectively. The K^+^/Na^+^ ratio increased from 1.0 under SAS to 2.2 under SAS+MT.

SAS increased proline and soluble sugar contents by 311.1% and 77.8%, respectively, compared with CK (Figure 5E,F), whereas soluble protein content decreased by 27.2% (Figure 5G). Compared with SAS, SAS+MT decreased proline and soluble sugar contents, although both remained higher than those in CK, while soluble protein content increased by 25.3%.

### 3.6. Oxidative Damage Indicators and Antioxidant Enzyme Activities

**Figure 6:**
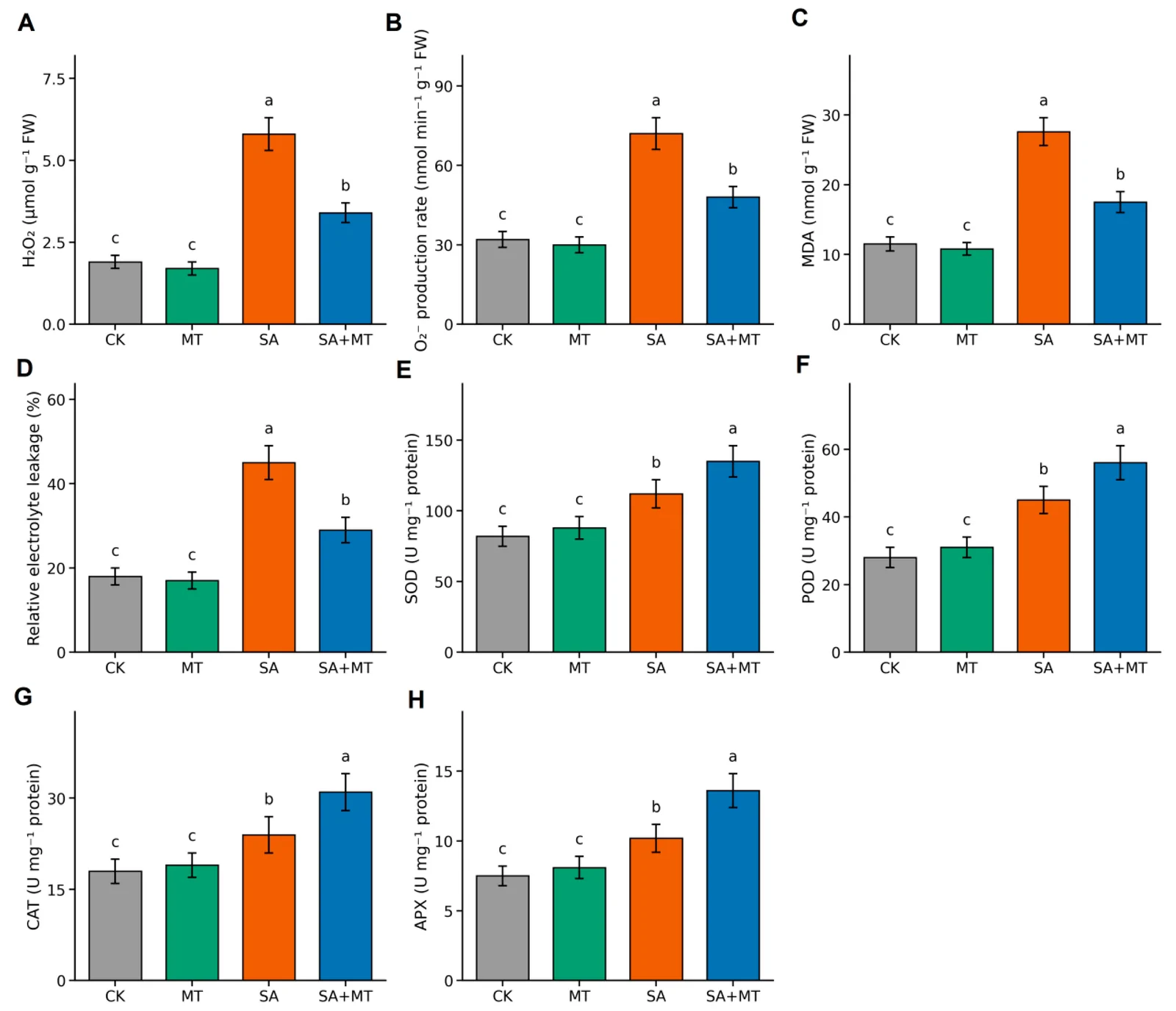
Oxidative damage indicators and antioxidant enzyme activities of pakchoi under different saline–alkali stress and melatonin treatments. Values of (**A**) H_2_O_2_ content, (**B**) O_2_^−^ production rate, (**C**) MDA content, (**D**) relative electrolyte leakage, (**E**) SOD activity, (**F**) POD activity, (**G**) CAT activity, and (**H**) APX activity under the different treatments. CK, normal nutrient solution plus foliar solvent control; MT, normal nutrient solution plus foliar application of 100 μM melatonin; SAS, saline–alkali stress plus foliar solvent control; SAS+MT, saline–alkali stress plus foliar application of 100 μM melatonin. Values are means ± SD (*n* = 5). Different lowercase letters indicate significant differences among treatments according to Tukey’s HSD test at *p* < 0.05.

Compared with CK, SAS increased H_2_O_2_ content, O_2_^−^ production rate, MDA content, and relative electrolyte leakage by 205.3%, 125.0%, 140.0%, and 150.0%, respectively (Figure 6A–D). Compared with SAS, SAS+MT decreased these parameters by 41.4%, 33.3%, 36.6%, and 35.6%, respectively. In addition, compared with SAS, SAS+MT increased SOD, POD, CAT, and APX activities by 20.5%, 24.4%, 29.2%, and 33.3%, respectively (Figure 6E–H).

## 4. Discussion

### 4.1. Melatonin Alleviated Saline–Alkali-Induced Growth Inhibition by Improving Water Status and Photosynthetic Capacity

Saline–alkali stress markedly inhibited *pakchoi* growth, as shown by the decreases in plant height, shoot fresh weight, shoot dry weight, leaf area, and relative water content. These responses are consistent with the typical physiological effects of salt-related stress, including restricted water uptake, osmotic imbalance, ionic toxicity, and reduced carbon assimilation [1,2,3,4]. In *pakchoi* and related *Brassica rapa* crops, salt stress has also been reported to suppress growth, alter photosynthetic performance, and disturb ion distribution [5,6,7,8].

In the present study, melatonin partially restored growth traits and leaf relative water content under saline–alkali stress. This indicates that melatonin did not simply stimulate growth under normal conditions but mainly acted as a stress-mitigation regulator. Similar protective effects of exogenous melatonin have been reported in maize, cotton, oat, wheat, and rice under salt stress, where improved growth was associated with enhanced photosynthesis, better antioxidant capacity, and improved ionic balance [25,26,27,28,29]. Therefore, the growth recovery observed in SAS+MT plants was likely the integrated outcome of improved water retention, partial photosynthetic protection, improved ion homeostasis, and reduced oxidative injury.

The gas-exchange results support this interpretation. Saline–alkali stress markedly decreased P_n_, G_s_, and T_r_, whereas C_i_ increased. The simultaneous decline in P_n_ and increase in C_i_ suggests that the reduction in photosynthesis was not attributable solely to stomatal closure. Possible non-stomatal limitations include a reduced biochemical capacity for CO_2_ assimilation and impairment of chloroplast photochemical processes and photosynthetic electron transport [3,4,37]. In the present study, this interpretation was supported by the accompanying decreases in F_v_/F_m_, Y_(II)_, ETR, qP, ET_o_/RC, and PI_ABS_, together with increases in V_J_, W_K_, and DI_o_/RC under SAS. These fluorescence responses indicate that PSII photochemical efficiency and electron transport were impaired under saline–alkali stress and may therefore have contributed to the observed non-stomatal limitation of photosynthesis. Melatonin reduced the stress-induced declines in P_n_ and G_s_ and lowered C_i_ relative to SAS, consistent with partial recovery of the coordination between CO_2_ diffusion and carbon assimilation. This response agrees with previous studies showing that melatonin can improve photosynthetic performance and protect photosynthetic machinery under salinity stress [25,27,30,31,32,33].

### 4.2. Melatonin Partially Maintained PSII Photochemical Activity and Electron Transport Under Saline–Alkali Stress

PSII is one of the most stress-sensitive components of the photosynthetic apparatus. In the present study, saline–alkali stress reduced F_v_/F_m_, Y_(II)_, ETR, and qP, while increasing NPQ. These changes indicate lower PSII maximum efficiency, reduced effective photochemical efficiency, restricted electron transport, lower reaction-center openness, and greater thermal energy dissipation. Chlorophyll fluorescence parameters are widely used to diagnose PSII photoinhibition and stress-induced changes in photochemical energy use [9,10,38].

The OJIP transient and JIP-test data provided additional evidence for PSII impairment. Under SAS, V*_J_* and W*_K_* increased, indicating acceptor-side limitation and possible donor-side or oxygen-evolving-complex disturbance. Meanwhile, ABS/RC and DI_o_/RC increased, whereas ET_o_/RC and PI_ABS_ decreased. These responses suggest that saline–alkali stress increased apparent excitation pressure per active reaction center, reduced electron transport per reaction center, and shifted more absorbed energy toward dissipation. JIP-test parameters are useful for separating stress effects on energy absorption, trapping, electron transport, and dissipation [11,12,13,14]. Therefore, the decrease in PI*_ABS_* under SAS indicates a broad reduction in PSII functional performance rather than inhibition at a single step.

Melatonin moderated these stress-induced changes. In SAS+MT plants, Y_(II)_, ETR, qP, ET_o_/RC, and PI_ABS_ were higher than in SAS plants, whereas NPQ, V*_J_*, W*_K_*, and DI_o_/RC were lower. This pattern suggests that melatonin was associated with improved PSII electron transport and lower excessive energy dissipation under saline–alkali stress. Previous studies have shown that melatonin can support photosynthetic electron transport and alleviate photoinhibition by regulating redox balance and protecting photosynthetic components under salt stress [30,31,32,33]. The present results extend this evidence to *pakchoi* under mixed saline–alkali stress and indicate that PSII functional maintenance is an important component of melatonin-mediated stress alleviation.

### 4.3. Improved Ion Homeostasis Was a Key Component of Melatonin-Mediated Saline–Alkali Stress Alleviation

Ion imbalance is a major cause of salt-related injury. Excessive Na^+^ accumulation disrupts enzyme activity, membrane function, nutrient uptake, and cellular metabolism, whereas K^+^ retention is essential for osmotic regulation and metabolic stability [3,15,17]. In this study, saline–alkali stress greatly increased Na^+^ content and reduced K^+^ and Ca^2+^ contents, resulting in a sharp decrease in the K^+^/Na^+^ ratio. This confirms that ionic toxicity and nutrient imbalance were major components of saline–alkali injury in *pakchoi*.

Melatonin reduced Na^+^ accumulation and increased K^+^ and Ca^2+^ contents under saline–alkali stress. As a result, the K^+^/Na^+^ ratio was substantially improved in SAS+MT plants compared with SAS plants. Maintenance of a high K^+^/Na^+^ ratio is closely related to salt tolerance because K^+^ supports enzyme activation, osmotic balance, and membrane potential, whereas excessive Na^+^ interferes with cellular metabolism [18,19,20]. Similar melatonin-induced improvements in Na^+^/K^+^ homeostasis have been observed in rice, wheat, and maize under salt stress [28,29,32].

The recovery of Ca^2+^ content may also be important. Calcium contributes to membrane stabilization, stress signaling, and ionic balance, and Ca^2+^ depletion can aggravate salt-induced membrane injury [2,4]. The higher Ca^2+^ content in SAS+MT plants was consistent with lower electrolyte leakage and reduced oxidative damage. Therefore, the ion-regulatory role of melatonin in this study was not limited to Na^+^ restriction but was associated with broader cation balance under saline–alkali stress.

An additional consideration is the potential contribution of Cl^−^ to the observed saline–alkali injury. Because NaCl was one component of the mixed stress treatment, Cl^−^ was supplied simultaneously with Na^+^ and may also have contributed to ionic stress. Experimental studies separating the effects of Na^+^ and Cl^−^ have shown that excessive Cl^−^ accumulation can independently suppress plant growth and photosynthetic capacity and can adversely affect PSII photochemical performance [50,51]. In the present study, however, Cl^−^ content was not quantified. Therefore, although the measured changes in Na^+^, K^+^, Ca^2+^, and the K^+^/Na^+^ ratio demonstrate substantial disruption of cation homeostasis under SAS, the relative contributions of Na^+^ and Cl^−^ toxicity cannot be distinguished from the present data. Future studies should quantify Cl^−^ accumulation and tissue distribution to determine whether melatonin also influences Cl^−^ homeostasis under mixed saline–alkali stress.

Osmotic adjustment also contributed to the stress response. SAS markedly increased proline and soluble sugar contents, indicating stress-related osmotic adjustment. However, SAS+MT plants had lower proline and soluble sugar levels than SAS plants but higher soluble protein content. This does not indicate weaker tolerance; rather, it suggests that melatonin reduced stress severity while maintaining metabolic stability. Under severe stress, excessive proline accumulation often reflects injury intensity, whereas moderate osmotic adjustment combined with better water status may be more favorable for growth recovery [3,4,39].

### 4.4. Melatonin Reduced Oxidative Damage by Strengthening Antioxidant Defense

Saline–alkali stress caused strong oxidative damage in *pakchoi* leaves, as shown by increased H_2_O_2_, O_2_^−^ production, MDA content, and relative electrolyte leakage. These indicators reflect ROS accumulation, lipid peroxidation, and loss of membrane integrity. ROS accumulation is a common consequence of salt stress because ionic toxicity, osmotic stress, and impaired photosynthetic electron transport increase electron leakage and oxidative injury [2,52].

Melatonin significantly reduced H_2_O_2_, O_2_^−^ production, MDA, and electrolyte leakage under saline–alkali stress. This protective effect was associated with increased SOD, POD, CAT, and APX activities in SAS+MT plants. SOD catalyzes the conversion of O_2_^−^ to H_2_O_2_, whereas CAT, POD, and APX participate in H_2_O_2_ detoxification [46,47,48,49]. The simultaneous enhancement of these enzymes indicates that melatonin improved enzymatic ROS-scavenging capacity and reduced membrane injury. Similar effects have been reported in other crops, where melatonin improved salt tolerance by enhancing antioxidant defense and reducing oxidative damage [21,23,24,25,26,27,28].

The relationship between antioxidant defense and photosynthetic protection is important. Saline–alkali stress can impair PSII electron transport, increasing ROS formation, which can further damage chloroplast membranes and photosynthetic proteins. This feedback intensifies photoinhibition and growth suppression. Melatonin likely interfered with this damaging cycle by enhancing antioxidant enzyme activity and reducing ROS accumulation. The concomitant recovery of ETR, PIABS, and Pn in SAS+MT plants supports this interpretation.

### 4.5. Integrated Physiological Interpretation of Melatonin-Mediated Saline–Alkali Stress Alleviation in Pakchoi

The present results support an integrated physiological interpretation in which melatonin partially alleviated saline–alkali stress through coordinated physiological responses involving photosynthesis, ion balance, osmotic adjustment, and antioxidant defense. First, melatonin helped maintain photosynthetic capacity by maintaining pigment contents, gas exchange, PSII photochemical efficiency, and electron transport. This was reflected by higher P_n,_ F_v_/F_m_, Y_(II)_, ETR, qP, ET_o_/RC, and PI*_ABS_* in SAS+MT than in SAS.

Second, melatonin improved ion homeostasis by reducing Na^+^ accumulation and increasing K^+^, Ca^2+^, and the K^+^/Na^+^ ratio. These changes can reduce ionic toxicity and support membrane stability, stomatal function, and metabolic activity. Third, melatonin enhanced antioxidant defense, as indicated by higher SOD, POD, CAT, and APX activities and lower ROS accumulation, MDA content, and electrolyte leakage. Fourth, melatonin helped maintain osmotic and metabolic stability by regulating proline, soluble sugar, and soluble protein levels.

These processes are functionally linked. Improved ion balance can reduce osmotic and ionic injury and support chloroplast function. More efficient PSII electron transport can reduce excess excitation pressure and limit ROS production. Stronger antioxidant defense can remove ROS and maintain membrane integrity. Improved osmotic adjustment can support leaf water status and cellular stability. Together, these coordinated responses may have contributed to the improved growth and water status observed in SAS+MT plants.

An important feature of the present experimental design is that saline–alkali stress and melatonin were initially imposed on different plant compartments. The mixed saline–alkali treatment was supplied through the root zone, whereas melatonin was applied foliarly. Therefore, the roots were directly exposed to the combined osmotic, ionic, and high-pH challenge, and root dysfunction could have contributed to the subsequent changes in shoot water status, ion balance, and photosynthetic performance. In contrast, foliar melatonin was associated with improved shoot physiological performance despite continued root-zone stress. Previous studies have shown that exogenous melatonin can improve root ion homeostasis and root physiological status under salt stress, including enhanced Na^+^ efflux and K^+^ influx [29,53]. However, root responses were not measured in the present study. Consequently, it remains unclear whether foliar melatonin directly or indirectly improved root function, and the observed changes in shoot ion contents cannot be attributed specifically to altered root uptake, root-to-shoot transport, or shoot-level ion partitioning.

However, this study was based on short-term physiological and biochemical endpoints and focused primarily on shoot responses. Root biomass, root architecture, root viability, root oxidative status, root ion fluxes, and tissue-specific ion distribution were not measured. The study also did not assess melatonin signaling genes, ion transporter expression, PSII protein composition, chloroplast ultrastructure, or long-term yield. Therefore, the present results demonstrate an integrated shoot physiological response to foliar melatonin under root-zone saline–alkali stress but do not establish whether melatonin directly protected the root system or define the underlying root-to-shoot signaling and transport mechanisms.

## 5. Conclusions

This study showed that mixed saline–alkali stress strongly inhibited pakchoi plant growth by disrupting leaf water status, photosynthetic activity, ion homeostasis, and oxidative balance. Under saline–alkali stress, pakchoi plants showed reduced biomass accumulation, pigment content, gas exchange, PSII photochemical efficiency, and OJIP-derived electron transport performance. These changes were accompanied by excessive Na^+^ accumulation, a sharp decline in the K^+^/Na^+^ ratio, increased ROS production, membrane lipid peroxidation, and electrolyte leakage.

Exogenous melatonin partially alleviated these adverse effects. Compared with SAS, SAS+MT increased P_n_, Y_(II)_, ETR, qP, ET_o_/RC, PI*_ABS_*, K^+^ content, Ca^2+^ content, K^+^/Na^+^ ratio, and antioxidant enzyme activities, while reducing Na^+^ accumulation, NPQ, V*_J_*, W*_K_*, DI*_o_*/RC, H_2_O_2_, O_2_^−^ production, MDA content, and relative electrolyte leakage. These results indicate that melatonin helped maintain PSII electron transport, improved ion homeostasis, strengthened antioxidant defense, and reduced membrane injury under saline–alkali stress.

Overall, melatonin functioned as a physiological regulator that improved the short-term tolerance of *pakchoi* plants to mixed saline–alkali stress. The protective effect was associated with coordinated regulation of photosynthetic electron transport, Na^+^/K^+^/Ca^2+^ balance, osmotic adjustment, and ROS-scavenging capacity. Further studies should examine root growth and viability, root ion fluxes, tissue-specific Na^+^, K^+^, Ca^2+^, and Cl^−^ distribution, melatonin-responsive ion transporters, chloroplast ultrastructure, and yield performance under longer-term saline–alkali conditions.

## Data Availability

The original contributions presented in the study are included in the article. Further inquiries can be directed to the corresponding author.

## Abbreviations

**Abbreviation**	**Full term**
CK	control treatment
MT	melatonin
SAS	saline–alkali stress
SAS+MT	saline–alkali stress plus melatonin
ROS	reactive oxygen species
RWC	relative water content
FW	fresh weight
TW	turgid weight
DW	dry weight
PPFD	photosynthetic photon flux density
SPAD	Soil Plant Analysis Development
Pn	net photosynthetic rate
Gs	stomatal conductance
Ci	intercellular CO_2_ concentration
Tr	transpiration rate
WUE	water-use efficiency
PSI	photosystem I
PSII	photosystem II
Fo	minimum fluorescence of dark-adapted leaves
Fm	maximum fluorescence of dark-adapted leaves
Fs	steady-state fluorescence
Fm′	maximum fluorescence under actinic light
F_v_/F_m_	maximum quantum efficiency of PSII photochemistry
Y_(II)_	effective quantum yield of PSII
ETR	electron transport rate
qP	photochemical quenching coefficient
NPQ	non-photochemical quenching
PAM	pulse-amplitude modulation
OJIP	O–J–I–P chlorophyll a fluorescence transient
JIP-test	analysis of OJIP fluorescence transients
VJ	relative variable fluorescence at the J step
WK	relative fluorescence associated with the K step
ABS/RC	absorption flux per active PSII reaction center
ET_0_/RC	electron transport flux per active PSII reaction center
DI_0_/RC	dissipated energy flux per active PSII reaction center
PIABS	performance index on an absorption basis
QA	primary quinone electron acceptor of PSII
ICP-OES	inductively coupled plasma optical emission spectrometry
MDA	malondialdehyde
REL	relative electrolyte leakage
SOD	superoxide dismutase
POD	peroxidase
CAT	catalase
APX	ascorbate peroxidase
EDTA	ethylenediaminetetraacetic acid
SD	standard deviation
ANOVA	analysis of variance
HSD	honestly significant difference
